# Translation, cross‐cultural adaptation and validation of the traditional Chinese Food Allergy Quality of Life‐Parental Burden questionnaire into simplified Chinese for use in mainland China

**DOI:** 10.1002/nop2.1807

**Published:** 2023-05-11

**Authors:** Zeen Li, Tingfan Leung, Lang Tian, Haiyan Liu, Wei He, Siyuan Tang, Qirong Chen, Guiyun Wang

**Affiliations:** ^1^ School of Nursing, Shandong Xiehe University Jinan Shandong China; ^2^ Department of Paediatrics The Chinese University of Hong Kong Hong Kong China; ^3^ Prince of Wales Hospital Hong Kong China; ^4^ Third Xiangya Hospital Central South University Changsha Hunan China; ^5^ Shandong Xiehe University Jinan Shandong China

**Keywords:** caregiver burden, children, food hypersensitivity, measurement property, quality of life

## Abstract

**Aim:**

The study aimed to translate and adapt the traditional Chinese Food Allergy Quality of Life‐Parental Burden Questionnaire (TC‐FAQL‐PB) into simplified Chinese language and determine the validity and reliability of the translated version.

**Design:**

A methodologic study design involving instrument translation and psychometric evaluation was used for the present study.

**Methods:**

The simplified Chinese FAQL‐PB (SC‐FAQL‐PB) was developed following Guillemin's guidelines for cross‐cultural adaptation. A convenience sample of 230 participants was recruited. The psychometric properties were examined using internal consistency, test–retest reliability, item discrimination, content validity and construct validity.

**Results:**

The values of I‐CVI ranged from 0.83 to 1.00. The CFA model revealed that the study supported the two‐factor model. The questionnaire had good internal consistency with a Cronbach's alpha coefficient of 0.946. The item‐total correlation values ranged from 0.707 to 0.866. Test–retest reliability showed that the intraclass correlation coefficient was 0.926 (95% CI, 0.830–0.968).

## 1 INTRODUCTION

Food allergy (FA), a common allergic disease in childhood, is the hypersensitive response by the immune system to proteins in food that can affect respiratory and cardiovascular systems, the gastrointestinal tract and the skin simultaneously and with a variety of severity (Pyrhönen et al., 2015). Children with FA often have multiple allergic comorbidities, such as asthma, eczema and hay fever (Gore et al., 2016). FA could be immunoglobulin (Ig)E‐mediated, non‐IgE‐mediated or mixed type (Lopez et al., 2022). Food intolerances were excluded from this study (Bischoff, 2022).

FA has been one of the fastest‐growing public health concerns with epidemic‐like features (Lack et al., 2016; Motosue et al., 2018; Mullins, 2007; Sicherer et al., 2000). In recent decades, FA involves up to 8% of children in the western world (Muraro et al., 2022), and the prevalence of FA in children has increased in western countries (Gupta et al., 2018; Lyons et al., 2020; Peters et al., 2017). A meta‐analysis showed that the prevalence of FA in children in mainland China—similar to that in western countries—is high (95% CI, 5.3%–6.5%) and increasing gradually (Wang et al., 2022). In an epidemiological survey conducted in three Chinese cities, the prevalence was ensured by the diagnosis of FA after taking detailed medical histories and involved skin prick tests and oral food challenges (OFC). The prevalence rate of FA among children aged 0–2 years was 5.6%–7.3%, and it also showed a gradual increase in the past 20 years (Ma, 2021; Ma et al., 2021).

For younger children with FA, the disease burden mainly falls on parents in several dimensions: physical burden, social burden, financial burden and psychological burden (Chooniedass et al., 2018; Golding, Gunnarsson, et al., 2021; Pitchforth et al., 2011). Parental burden in this study was defined as the negative experiences in physical, psychological, social and economic aspects that parents may undergo in the process of taking care of their children (Chou, 2000). Parental burden emphasized the multi‐dimensional impact of care on parents. FA requires strict avoidance of food allergens and is now the main method of food allergy treatment (Chapman et al., 2006, “EAACI Food Allergy and Anaphylaxis Guidelines: diagnosis and management of food allergy,”, 2014, Urisu et al., 2011). The elimination of allergens is not simple, as many products contain ‘hidden ingredients’ or traces of food with allergens such as nuts (Peniamina et al., 2014). Hence, FA brought an extra burden on parents because they need to spend more time and effort on grocery shopping as avoidance demands and have more frequent mealtime concerns than those without FA (Linda et al., 2017). FA also brought a social burden to parents, with more disruptions in daily activities and restrictions in social activities (Broome et al., 2015; Chooniedass et al., 2018; Golding, Gunnarsson, et al., 2021). In terms of financial burden, families who raised a child with FA also had greater financial costs than those without FA (Golding, Simons, et al., 2021). With respect to psychological burden, they had greater stress and worry and lower levels of confidence in their ability to take care of their children and keep them safe (Broome et al., 2015; Golding, Gunnarsson, et al., 2021). Many parents, especially those with a child with life‐threatening food allergies, were afraid of the lack of management information and the threat of accidental exposures that necessitated vigilance (Broome et al., 2015). These burdens in different dimensions described above indicated that they indeed negatively affected the health‐related quality of life (HRQL) of parents (Chen, Li, et al., 2022; Stensgaard et al., 2017). Another scoping review found that parents with a child with FA reported reduced quality of life in at least one area, such as physical health, psychological health, social relationships, etc., because of the heavy burden that FA had brought into their lives (Golding, Gunnarsson, et al., 2021). Hence, more attention should be paid to the parental burden of parents having a child with FA (Chooniedass et al., 2018; Golding, Gunnarsson, et al., 2021; Pitchforth et al., 2011).

Although qualitative research can be used by health professionals to measure parental burden more comprehensively and personalized, considering the limitations of manpower and time in clinical practice, a reliable quantitative tool is also necessary to evaluate the parental burden more quickly amongst parents having a child with FA. Instruments for measuring parental burden include general measures and disease‐specific measures. General measures such as the Zarit caregiver burden interview (ZBI) and caregiver burden inventory (CBI) are applied for measuring the general burden of caregivers including the parents of children. However, these instruments have not been used in assessing the concrete burden of parents of children with FA because of limited focus and scope.

The Food Allergy Quality of Life‐Parental Burden Questionnaire (FAQL‐PB) was a disease‐specific instrument developed by Cohen et al. and can be used to assess the burden of parents of children aged 0–18 years old with FA (Cohen et al., 2004). There are 17 items covering family and social activities, meal preparation, health issues and emotional issues. Higher scores reflect a greater parental care burden. The total score ranges from 17 to 119. The original English version has been translated into many languages and validated in the United Kingdom, Australia, Thailand, Iran, South Korea and Hong Kong, China. All the versions were validated with satisfactory reliability and validity (Allen et al., 2015; Cohen et al., 2004; Fathi et al., 2016; Knibb & Stalker, 2013; Leung et al., 2009; 이 et al., 2018). The FAQL‐PB questionnaire was translated into a Chinese version (the traditional Chinese FAQL‐PB or TC‐FAQL‐PB) by Professor TF Leung in 2009; it was mostly used in Hong Kong, with satisfactory psychometric properties (e.g. internal consistency—Cronbach's *α* = 0.976, test–retest reliability—Intraclass correlation coefficient (ICC) value was 0.701 (95% CI, 0.654–0.748, *p* < 0.001) (Leung et al., 2009; Streiner, 2003). Exploratory factor analysis extracted two principal components: Items 1–3 belonging to the domain of ‘limitation in life’ and items 4–17 belonging to the domain of ‘emotional distress’ (Leung et al., 2009).

Considering the writing reform initiated by the central government of the People's Republic of China in 1956 for easing the learning process in both recognition (reading) and production (writing), today the majority of people in mainland China use the simplified script. Hence, some people in mainland China, especially those in low‐income areas, may not understand the traditional Chinese characters (Liu et al., 2016). In addition, when we chose parents in mainland China to fill in the TC‐FAQL‐PB questionnaire (developed by Professor TF Leung) in the pre‐survey, they generally reported that they could not read and understand the traditional Chinese characters easily. Regarding cultural differences, some wordings in the traditional Chinese language are not applicable in the simplified Chinese language because of the differences in semantics, idiomatic expressions and concepts (Beaton et al., 2001, 2007; Guillemin et al., 1993). Hence, TC‐FAQL‐PB, which is in the traditional Chinese language, could not be used widely in mainland China. As such, the FAQL‐PB in simplified Chinese language (SC‐FAQL‐PB) is required for measuring the parental burden of parents having a child with FA in mainland China.

As the simplified Chinese language and the traditional Chinese language both belong to the Chinese language system (Liu et al., 2016) and the TC‐FAQL‐PB was validated with good psychometric properties, this study aimed to translate the TC‐FAQL‐PB into the simplified Chinese language and evaluate the psychometric properties of SC‐FAQL‐PB in mainland China. It will bring a disease‐specific, valid and reliable instrument for health professionals to measure the parental burden and evaluate the effect of corresponding intervention in alleviating the parental burden of parents having a child with FA in mainland China.

## 2 METHODS

### 2.1 Study design

A methodologic study design involving instrument translation and psychometric evaluation was used. We finally obtained the SC‐FAQL‐PB through a two‐step process: (1) translation and cultural adaptation and (2) psychometric analysis. A cross‐sectional validation survey was used to evaluate the psychometric properties of the questionnaire.

### 2.2 Translation and cultural adaptation

The translation and cultural adaptation in this study were done with prior permission and according to the Guillemin guidelines (Guillemin et al., 1993), as recommended by Beaton et al. in the ‘cross‐cultural adaptation of self‐report measures’ (Beaton et al., 2001, 2007; Guillemin et al., 1993). The process involving translation, backward translation, expert committee review, pre‐testing and weighting of scores (it was not necessary for all the translations) adds the value of resolving translation differences and helps produce a single consensus‐based translation (see Table S1). Considering back translation is generally used to translate back from the final language into the source language to help improve the quality of the final version, for our translation, it was not necessary to perform a back translation because there was a one‐to‐one correspondence between the traditional and simplified Chinese characters. The TC‐FAQL‐PB is a 7‐point Likert questionnaire, and it has been validated with good psychometric properties in Hong Kong; hence, this study did not consider adapting the weights of scores (Guillemin et al., 1993). We completed the translation and cultural adaption through the following three steps:

#### 2.2.1 Translation

This translation process will focus on the conversion from the traditional Chinese characters to simplified characters. The simplified Chinese evolved from the traditional Chinese language and they all belong to the Chinese language system (Liu et al., 2016), and there was a one‐to‐one correspondence between traditional and simplified Chinese characters. For example, the traditional Chinese script ‘討’ can only be translated into ‘讨’ in the simplified Chinese script. Considering back translation is used to translate back from the final language into the source language to help improve the quality of the final version, there was no such need for our translation because the simplified Chinese script “讨” can only be translated to “討” because of the one‐to‐one corresponding between them. In this study, first, a Chinese‐speaking researcher translated the items of FAQL‐PB from traditional Chinese into simplified Chinese. Second, a nursing master's student who is a Macao native and fluent in reading simplified Chinese verified the translation. Then, we prepared the first version of the SC‐FAQL‐PB.

#### 2.2.2 Committee review

To ensure that the translation is fully comprehensible and to evaluate the equivalence of items between the two versions of the questionnaire, including semantic equivalence, cultural equivalence, experiential equivalence and conceptual equivalence, five experts were invited to form an expert committee to do the cross‐cultural adaptation of the questionnaire (Beaton et al., 2001, 2007, Guillemin et al., 1993). The eligibility criteria for the experts were as follows: (a) those with a master's degree or above, (b) those with expertise in paediatric care and psychology and (c) those well acquainted with the structural aspects of instrument construction. During this process, a clear consensus regarding the wording, suitability and equivalence of the questionnaire was reached. More details about the equivalence of the questionnaire are shown in Table S1. This step generated the second version of the SC‐FAQL‐PB. Then, the TC‐FAQL‐PB's author was required to review the second version of the SC‐FAQL‐PB. The third version of the SC‐FAQL‐PB was generated based on the comments.

#### 2.2.3 Pre‐testing

A pilot test of the third version of the SC‐FAQL‐PB was further administered to 10 additional parents of children with FA to estimate the comprehensibility of each item. The wording of the items was adjusted after the pilot study to make it more comprehensible. Subsequently, the fourth version of the SC‐FAQL‐PB to be validated was completed.

### 2.3 Psychometric analysis

#### 2.3.1 Sample and data collection

Given that FA tends to commonly occur in early childhood, first occurring before the age of 5 years (Cui, 2021), convenience sampling was used to recruit the parents of children aged 0–5 years who were diagnosed with FA. Inclusion criteria were as follows: (1) a parent aged ≥18 years who (2) had at least one child aged ≤5 years who were clinically diagnosed as having FA by paediatricians and immunologists (diagnosis could have been made via OFC, skin prick test, spot patch test, serum IgE detection or dietary avoidance), (3) was the main caregiver of children and (4) could understand and communicate normally. The exclusion criteria were as follows: (1) a parent whose child had experienced complications with malignant diseases or other organic lesions and (2) a parent who had a mental illness or cognitive impairment. Here, mental illnesses referred to severe mental illnesses leading to the inability of filling in the questionnaire, for example, mental disorders, which had substantial impairments of social and cognitive functions (Ochneva et al., 2022). The cognitive impairment referred to patients having trouble with memory, paying attention, speaking or understanding, and they might have difficulty recognizing people, places or things. (Gobom et al., 2022).

Based on the Kendall sample estimation method (Lu & Fang, 2003), the sample size should be 5–10 times larger than the number of items in the questionnaire. A 15% refusal rate and 10% invalid questionnaires were estimated to adjust the sample size. In addition, according to the principle of the minimum sample size of 200 for confirmatory factor analysis (Nicholls et al., 2015), an ideal sample size of 220 was set. To evaluate the test–retest reliability, the ICC value was used to show the ratio of the intra‐individual variance and the inter‐individual variance (Kiotseridis et al., 2011). According to Bujang's review (Souza‐Rua et al., 2019) for measuring the test–retest reliability, the minimum sample size of 15 subjects was recommended because we expected to achieve the ICC value of at least 0.60 as the minimum acceptable value (Souza‐Rua et al., 2019). Considering the issue of memory recall after filling in the questionnaire and the stability of the measured variables, 10 days was selected as the time span (Souza et al., 2017). Hence, we chose 22 parents to perform the retest 10 days after the first distribution. Finally, we distributed 254 questionnaires to parents of children diagnosed with FA. A total of 230 completed questionnaires were collected, achieving a response rate of 90.55% (230/254). All 22 participants provided the retest 10 days after the first distribution.

Data collection began in September 2021 and lasted for 3 months. We recruited the participants through the Internet and the child health centres of three tertiary hospitals in Changsha, Hunan Province, China. There was no significant difference between participants recruited via these two methods. All the participants needed to fill in the online questionnaires via Wenjuanxing, a questionnaire collection platform used in mainland China (Chen, Zhou, et al., 2022; Li, Ji, & Sun, 2022). For the recruitment of participants through the Internet, we used WeChat (an online social software used in mainland China) to find some chat groups of parents of children with FA and sent them the online questionnaire (She et al., 2022). Before filling in the questionnaire, they were asked whether their children had been diagnosed with FA by paediatricians and immunologists. If they answered ‘no’, they would automatically be withdrawn from the survey. The parents were informed of the research‐related information, including the study goals, investigation process, anonymity and voluntary participation before filling in the questionnaire, and if they answered ‘consent to participate’ and provided electronic signatures, they would continue to fill in the questionnaire. Wenjuanxing ensured that the respondents completed the questionnaire to avoid missing data. It also avoided the problems of manual data entry and improved the efficiency and accuracy of data collection and data integration. Those who had difficulty filling in the online questionnaire were allowed to contact the researchers to guide them to finish or ask for the paper‐based questionnaire. Researchers' contact information was provided at the time of recruitment.

#### 2.3.2 Measures

The SC‐FAQL‐PB comprised 17 items. The 17 questions were each 7‐point Likert items, and the index was scored from one ‘not troubled’ to seven ‘extremely troubled’, with a higher FAQL‐PB score indicating a higher burden. The steps of translation and cultural adaptation have been reported above.

In addition to the SC‐FAQL‐PB questionnaire, children's and parents' demographic information and other disease‐specific characteristics of children with FA were also collected. The children's information included age in months, gender, corresponding syndromes, frequency of food allergies reported, types of allergen, whether they had experienced severe anaphylaxis and whether they had visited the emergency room. Parents' information included gender, age, family history of self‐reported allergies, education level, working conditions and knowledge about FA (self‐reported). The information about these characteristics was used as identification information to help collect a more representative sample to test the SC‐FAQL‐PB. Table 1 summarizes the demographic characteristics and FA‐related information for parents and their children. The majority of the questionnaires (96.1%, 221/230) were completed by the mothers of children with FA. The average age was 30.0 years (SD = 3.93) and most (64.8%) were employed. Children were mainly allergic to cow's milk (77.8%), eggs (47.4%), peanuts (17.0%), fish (16.1%) and shrimp (19.6%). Most children were sensitive to more than one food allergen (92.6%, 213/230). Symptoms included cutaneous signs (57.4%, 132/230) (e.g. facial swelling, urticaria, itching, rash, etc.); respiratory symptoms (30.0%, 69/230) (e.g. sneezing, runny nose, cough, dyspnoea, etc.) and gastrointestinal symptoms (63.0%, 145/230) (e.g. gaseous distention, gastroesophageal reflux, vomiting, diarrhoea, haematochezia, etc.).

**TABLE 1:** Characteristics for parents and their children (*n* = 230).

Variable	Values/*N*(%)
Parent gender	
Female	221(96.1%)
Male	9 (3.9%)
Age of parents (mean years)	30 ± 3.93
Education level	
Junior high school or below	12 (5.2%)
Senior high school	28 (12.2%)
College	95 (41.3%)
Undergraduate or above	95 (41.3%)
Working conditions	
Holding a job	149 (64.8%)
Not on work	81 (35.2%)
Family history of self‐reported FA	
Father	62 (27.0%)
Mother	66 (28.7%)
Both of parents	26 (11.3%)
Having knowledge about food allergy (self‐reported)	70 (30.43%)
Food‐allergic children's age (mean months)	
0–5 months	16 (7.0%)
6–11 months	59 (25.7%)
12–23 months	144 (62.6%)
24–35 months	8 (3.5%)
36–47 months	2 (0.9%)
48–59 months	1 (0.4%)
Gender of child with FA	14.51 ± 6.67
Female	100 (43.5%)
Male	130 (56.5%)
Frequency of food allergens reported	
1 allergen	17 (7.4%)
≥2 allergens	213 (92.6%)
Types of allergen	
Cow's milk	179 (77.8%)
Egg	109 (47.4%)
Soybean	40 (17.4%)
Fish	37 (16.1%)
Shrimp	45 (19.6%)
Nut	21 (9.1%)
Peanut	39 (17.0%)
Having visited the emergency room	49 (21.3%)
Suffering from severe anaphylaxis	2 (0.9%)
Suffering from the corresponding syndrome	
Dermatological signs	132 (57.4%)
Gastrointestinal symptom	145 (63.0%)
Respiratory symptom	69 (30.0%)
Others	59 (25.7%)

#### 2.3.3 Statistical analysis

The IBM SPSS program version 26.0 and the AMOS 25.0 program were used for data analysis. The psychometric properties were examined using internal consistency, test–retest reliability, item discrimination, content validity and construct validity.

The internal consistency was assessed using Cronbach's alpha (*α*). Cronbach's α over 0.70 was recommended for the adequacy of reliability coefficients (Streiner, 2003). To evaluate the test–retest reliability, the ICC value was used to show the ratio of the intra‐individual variance and the inter‐individual variance (Kiotseridis et al., 2011). Critical ratio (CR) and item‐total correlation (ITC) were calculated to measure the discriminating effectiveness of an item. The higher the value, the more effective the item. The item was effective when the value of the CR was >3 and the ITC value was >0.4.

Two types of content validity index (CVI) were calculated to assess content validity, including item‐level CVI (I‐CVI) and scale‐level CVI (S‐CVI). S‐CVI contained the average S‐CVI (S‐CVI/Ave) and universal agreement S‐CVI (S‐CVI/UA). I‐CVI ≥0.78 and S‐CVI ≥0.9 were considered good content validity (Polit & Beck, 2010). Six experts in nursing and paediatrics were asked to provide appropriate scoring on a 4‐point Likert scale (1 meaning ‘not relevant’ to 4 meaning ‘highly relevant’) to evaluate the degree of relevance between each of the items and the conceptual framework and to provide their comments on the items. The eligibility of the experts was determined using the following criteria: (a) those with a master's degree or above, (b) those with expertise in paediatric care or/and psychology and (c) those well acquainted with the structural aspects of instrument construction.

Confirmatory factor analysis (CFA) was used to further explore the construct validity of the SC‐FAQL‐PB and verify the two‐factor structure proposed by Professor TF Leung (the TC‐ FAQL‐PB's author) (Leung et al., 2009). The CFA was conducted in AMOS 25.0. Fitting indices were used in the CFA to assess the goodness‐of‐fit of the model and the factor load value of each item. In this study, eight types of indices were used as follows: chi‐squared tests (*χ*2), root mean‐square residual (RMR), goodness‐of‐fit index (GFI), adjusted goodness‐of‐fit index (AGFI), normed fit index (NFI), comparative fit index (CFI), Tucker‐Lewis fit index (TLI) and root mean square error of approximation (RMSEA) (Marsh et al., 1998). This study defined an acceptable model fit: RMR ≤ 0.05, RMSEA ≤ 0.08, GFI, AGFI, NFI, TLI and CFI ≥ 0.90. Chi‐square (*χ* ^2^) was considered significant when *p* < 0.05 and *χ2/df* ≤3 (Hu & Bentler, 1999).

### 2.4 Ethical considerations

This research was approved by the relevant institutional Ethical Review Committee on 15 July 2021.

### 2.5 Patient and public involvement

The study was designed to translate the FAQL‐PB questionnaire into simplified Chinese and evaluate its psychometric properties. However, participants were not involved in the survey instrument, recruitment or the conduct of the study. Participants were kept anonymous in this study, and hence the study team will be unable to disseminate the results of the study to the participants.

## 3 RESULTS

### 3.1 Translation and cultural adaptation

**FIGURE 1:**
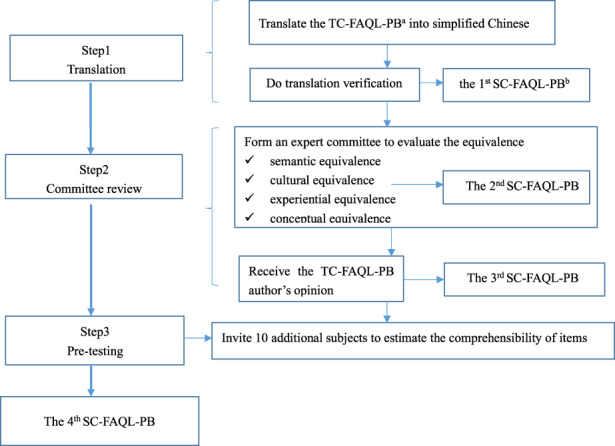
Instrument translation and cross‐cultural adaptation procedure of the SC‐FAQL‐PB*.Note: ^a^ TC‐FAQL‐PB: The traditional Chinese FAQL‐PB. ^b^ SC‐FAQL‐PB: The simplified Chinese FAQL‐PB. * The translation and cross‐cultural adaptation procedure were based on the Guillemin guidelines (Guillemin et al., 1993).

The specific translation procedure is presented in Figure 1, based on the Guillemin guidelines (Guillemin et al., 1993). The committee addressed several cross‐cultural adaptations in the first SC‐FAQL‐PB. We changed the wording of the TC‐FAQL‐PB about ‘food allergies’, ‘go to the restaurant’, ‘by the possibility’, etc., according to the semantic difference, idiomatic differences and conceptual differences. Then, we prepared the second SC‐FAQL‐PB. No changes were made when sending the second SC‐FAQL‐PB to Professor TF Leung, and the third SC‐FAQL‐PB was generated. The parents did not have major comments when filling in the third SC‐FAQL‐PB, and only minor changes in spelling were made by the researcher. Finally, we prepared the fourth SC‐FAQL‐PB based on the feedback and suggestions of the parents. More specific information about cultural adaptation, the TC‐FAQL‐PB (see Table S1) and the final version of the SC‐FAQL‐PB are presented in Table S2.

### 3.2 Psychometric analysis

The psychometric properties were examined with a convenience sample of 230 participants. All 22 participants provided the retest 10 days after the first distribution for test–retest reliability. The information about participants' characteristics proved the representativeness and diversity of the sample in this study. More details are shown in ‘Measures’ and Table 1.

#### 3.2.1 Reliability and item analysis

##### Reliability

The reliability results are listed in Table 2. Internal consistency was confirmed by Cronbach's alpha coefficients of 0.946 for the total questionnaire and 0.891–0.954 for the two dimensions (‘limitation in life’ and ‘emotional distress’); all the Cronbach's *α* coefficients were over 0.70, indicating a high level of internal consistency (Streiner, 2003). Although Cronbach's alpha of the total questionnaire of 0.946 was lower than the TC‐FAQL‐PB (Cronbach's *α* = 0.976), it also indicated the SC‐FAQL‐PB had an appropriate internal consistency (Leung et al., 2009).

**TABLE 2:** Cronbach's alpha and intra‐class correlation for test and retest for the SC‐FAQL‐PB (*n* = 230).

Category	Cronbach's alpha	ICC test–retest*
The total scales	0.946	0.926
Domain 1 (Limitation in life)	0.891	0.871
Domain 2 (Emotional distress)	0.954	0.927

* CI = 95%, *p* = 0.001.

The ICC value of the questionnaire was 0.926 (95% CI, 0.830–0.968) and that for the two dimensions ranged from 0.871 to 0.927. All the values were above 0.60, indicating the stability of the measure (Souza et al., 2017), and the 10‐day time span for testing and retesting adequately proved the reliability of the results (Souza‐Rua et al., 2019).

##### Item analysis

As presented in Table 3, CR values ranged from 6.237 to 21.832 with statistical significance (*p* < 0.01), and the ITC values ranged from 0.707 to 0.866, with statistical significance (*p* < 0.01). CR values were >3 and the ITC values were >0.4, indicating that all items on the SC‐FAQL‐PB had good discrimination.

**TABLE 3:** Critical ratio (CR), item‐total correlation (ITC) values and factor loading of the SC‐FAQL‐PB items (*n* = 330).

Item	CR value	ITC value	Factor loading^a^
1	6.237**	0.491**	0.78
2	7.855**	0.513**	0.87
3	9.119**	0.561**	0.92
4	13.379**	0.707**	0.70
5	13.703**	0.720**	0.69
6	26.846**	0.866**	0.89
7	19.434**	0.841**	0.86
8	14.437**	0.768**	0.75
9	14.982**	0.733**	0.74
10	18.661**	0.790**	0.79
11	17.842**	0.794**	0.77
12	21.623**	0.832**	0.85
13	15.572**	0.765**	0.78
14	15.514**	0.754**	0.76
15	14.384**	0.741**	0.74
16	12.090**	0.704**	0.66
17	21.832**	0.831**	0.83

^a^ Standardized factor loadings in the confirmatory factor analysis model of SC‐FAQL‐PB.

** *p*<0.01.

#### 3.2.2 Validity analysis

##### Content validity

Six experts consisting of two paediatric nursing educators, a psychiatric nursing educator, a senior nurse and two paediatricians were invited to evaluate the applicability of the items. Table S3 shows that the computed I‐CVI values ranged from 0.83 to 1.00, the average S‐CVI (S‐CVI/Ave) was 0.99 and universal agreement S‐CVI (S‐CVI/UA) was 0.94, indicating good content validity (Souza et al., 2017).

##### Construct validity

**FIGURE 2:**
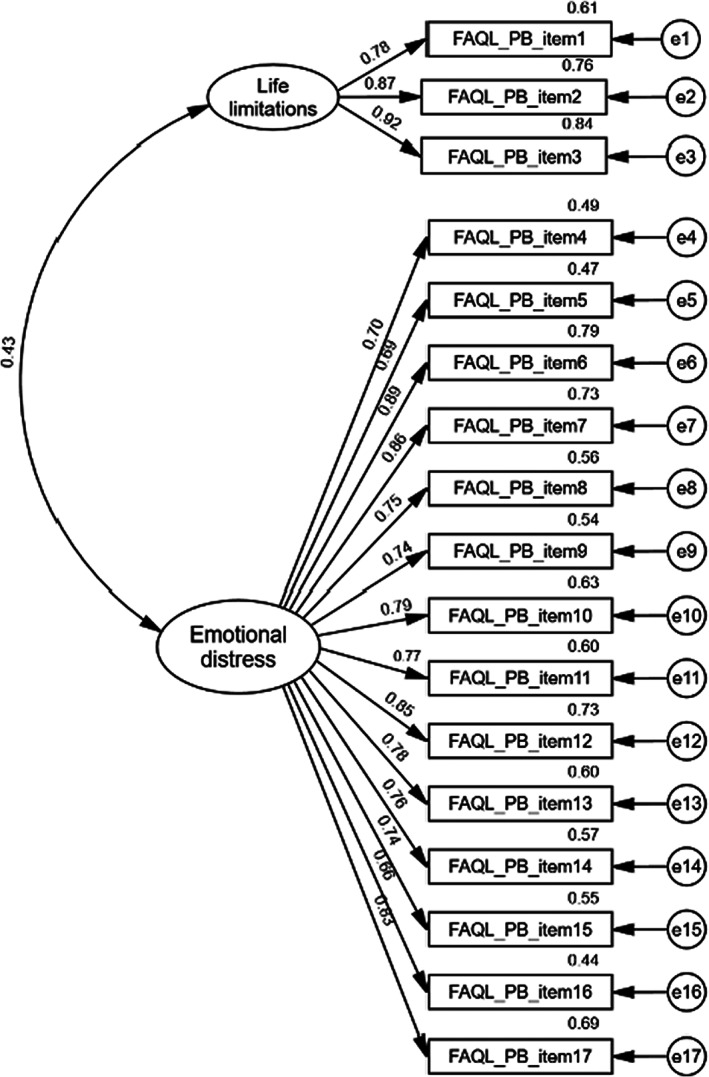
Factor loadings for the 17 items of the two‐factor structure.

The CFA model (Figure 2) was based on the two‐factor structure of the TC‐FAQL‐PB. Table 4 presents the results of the confirmatory factor analysis. The results were as follows: *χ* ^2^ */df* = 1.65, *p* < 0.01; RMSEA = 0.053; GFI = 0.908; AGFI = 0.880; NFI = 0.934; TLI = 0.969 and CFI = 0.973. All items had appropriate factor loadings ≥0.4, ranging from 0.66 to 0.92, which suggested that the overall data model fit in the current sample of Chinese mainland parents of children with FA. The details of the factor loadings of each item are shown in Table 3. The CFA results confirmed the two‐factor model as the most reasonable fit for explaining the factor construct of the SC‐FAQL‐PB: Items 1–3 were included in the factor ‘limitations in life,’ and Items 4–17 were included ‘emotional distress’.

**TABLE 4:** Goodness‐of‐fit indices for the two‐factor model in the confirmatory factor analysis^*^ (*n* = 230).

	RMSEA	GFI	AGFI	CFI	NFI	TLI	*χ* ^2^/*df*
Criterion	≤0.08	≥0.90	≥0.90	≥0.90	≥0.90	≥0.90	≤3
Result	0.053	0.908	0.880	0.973	0.934	0.969	1.65

Abbreviations: AGFI, adjusted goodness of–of–fit index; GFI, goodness of––of–fit index; RMSEA, root mean square error of approximation; TLI, Tucker–Lewis index.

* *p ≤* 0.001.

## 4 DISCUSSION

Parental burden refers to the people (parents having a child/children with FA), health (parents' mental health and social adaptation), environment (involving the physical, humanistic, social and ecological environment surrounding the human body) and nursing (health professionals need to evaluate the parental burden and take measures to relieve it), which involves the four basic concepts of nursing science (Li, Ji, Yang, & Xie, 2022). It indicates that focusing on parental burden was significant for nursing science. As FA has become one of the fastest‐growing public health problems with epidemiological characteristics (Mullins, 2007; Sicherer et al., 2000), a valid, disease‐specific instrument is needed to accurately measure the parental burden among parents having a child with FA.

While the FAQL‐PB has been used across the United Kingdom, Australia, Thailand, Iran, South Korea and Hong Kong, China to measure the parental burden among parents having a child with FA, this study was the first attempt to adapt the instrument for mainland China (Allen et al., 2015; Cohen et al., 2004; Fathi et al., 2016; Leung et al., 2009; 이 et al., 2018). Although there are 56 official ethnic groups in mainland China and some of them have their own language, all of them can read and write simplified Chinese scripts, especially young adults. Hence, the SC‐FAQL‐PB can be widely used in mainland China. As the simplified Chinese language evolved from the traditional Chinese language and they both belong to the same Chinese language system (Liu et al., 2016) and as both Hong Kong and mainland China share the same cultural roots although some specific cultural differences exist, it was rational and effective to directly translate the TC‐FAQL‐PB into simplified Chinese. The explicit translation and adaptation were based on the guideline for the cross‐cultural adaptation proposed by Guillemin and multiple translation strategies were used to obtain an appropriate quality and equivalent translated instrument (Beaton et al., 2007; Guillemin et al., 1993). In terms of the language, eligible translators translated the traditional Chinese scripts into simplified Chinese scripts. With respect to cultural aspects, considering the items of this questionnaire have excellent versatility, all the items remained, but some wordings in the traditional Chinese scripts were not applicable in simplified Chinese because of the differences in semantics, idiomatic expressions and concepts; hence, the panel experts modified some text in the items due to the cultural differences in the two contexts. The details are shown in Table S2.

The SC‐FAQL‐PB showed acceptable psychometric properties for measuring the parental burden for FA children in mainland China. It is a valid and valuable instrument that could be used in longitudinal research to help clinical professionals detect parental burden changes. It could be applied to diverse groups of parents using simplified Chinese to compare the variations in their burden. The methodology used in this study could provide experience and suggestions for other researchers who are interested in the translation and adaptation of scales.

### 4.1 Limitations

Several limitations of this study are acknowledged. First, given that FA tends to commonly occur in early childhood—first occurring before the age of 5 years (Cui, 2021)—and that parents of children aged 0–5 years are largely responsible for managing their child's FA and thus often experience a high parental burden, we recruited only parents with children aged 0–5 years who had been diagnosed with FA; hence, the general use of the questionnaire requires caution for other age groups. Second, we relied on parental reports on the diagnosis of FA. Nevertheless, we took several measures to ensure the diagnosis of FA was made by paediatricians and immunologists rather than the parents themselves; for example, the questionnaire asked for specific diagnosis methods, types of allergens, the name of the hospital, etc. Third, convenience sampling cannot guarantee sample diversity. For example, the sample in our study had very few parents with low levels of education. The reason may be that parents with low education levels may not pay as much attention to FA and have lower willingness to seek medical help; it could also be that there were fewer diagnoses of FA in low‐income areas. Hence, the questionnaire needs to be validated on parents with lower education levels. In addition, although the higher the score, the heavier the parental burden, the questionnaire did not specify any threshold to judge the degree of burden reasonably. Thus, more attention will be required to finding the appropriate thresholds in future studies. Finally, the translation and cultural adaptation were based on the TC‐FAQL‐PB, which was a traditional Chinese language version translated from the original FAQL‐PB questionnaire. As such, it may affect the consistency of the original questionnaire. However, questionnaire translation is time consuming and demands high requirements, and the TC‐FAQL‐PB has good psychometric properties in Hong Kong, China. Hence, we finally chose to translate the TC‐FAQL‐PB. In addition, we did expert consultation again in the study to ensure the content validity of the SC‐FAQL‐PB. The results of the psychometric analysis also showed that this questionnaire had good psychometric properties (e.g. internal consistency—Cronbach's *α* = 0.946, test–retest reliability—ICC value was 0.926 (95% CI, 0.830–0.968, *p* < 0.001), the CFA model revealed that the study supported the two‐factor model).

## 5 CONCLUSION

The SC‐FAQL‐PB questionnaire has been proven to be a valid and reliable measurement tool to capture the main features of Chinese parental burden among parents having a child with FA. It will help health professionals detect the associated factors with parental burden and make corresponding interventions in mainland China.

## AUTHOR CONTRIBUTIONS

Study design: ZL, QC, WH; Study supervision: ST, QC, TL, LT; Manuscript writing: ZL, QC, HL, TL, GW; Critical revisions for important intellectual content: TL, WH, HL, GW.

## FUNDING INFORMATION

The authors have not declared a specific grant for this research from any funding agency in the public, commercial or not‐for‐profit sectors.

## CONFLICT OF INTEREST STATEMENT

There is no interest in disclosure in this study.

## Supporting information


Table S1

Table S2

Table S3

## Data Availability

The data that support the findings of this study are available on request from the corresponding author. The data are not publicly available due to privacy or ethical restrictions.
