# Supplementary material for: Translation, cross‐cultural adaptation and validation of the traditional Chinese Food Allergy Quality of Life‐Parental Burden questionnaire into simplified Chinese for use in mainland China
Source: Nurs Open. 2023 May 11;10(8):5627–37. doi: 10.1002/nop2.1807 (PMC10333859; doi:10.1002/nop2.1807)
Supplement: Supplementary file 1 — Table S1 [file NOP2-10-5627-s002.docx]

**Table S1** The Traditional Chinese FAQL-PB (in traditional Chinese).

| 條目 | (1=沒有煩惱；2=些微煩惱；3=少許煩惱；4=有些煩惱；5=頗大煩惱；6=相當煩惱；7=嚴重煩惱) |
| --- | --- |
| 1. | 如你和你的家庭計劃假期，你所選擇的假期會因孩子患有食物敏感而有多少限制？ |
| 2. | 如你和你的家庭計劃去酒樓進膳，你所選擇的酒樓會因孩子患有食物敏感而有多少限制？ |
| 3. | 如你和你的家庭計劃參加他人舉辦而包括食物的社交活動（如派對、假期等），你因孩子患有食物敏感而對能夠參加這些包括食物的社交活動有多少限制？ |
| 4. | 在過往一星期，你因孩子患有食物敏感而對需要額外時間準備膳食（如閱讀標籤、額外購物時間、準備額外膳食等）感到多少煩惱？ |
| 5. | 在過往一星期，你因孩子患有食物敏感而對和孩子外出需要做的特別預備措施感到多少煩惱？ |
| 6. | 在過往一星期，你因孩子患有食物敏感而產生的緊張不安感到多少煩惱？ |
| 7. | 在過往一星期，你對孩子可能不能夠克服食物敏感而感到多少煩惱？ |
| 8. | 在過往一星期，你因孩子患有食物敏感而可能或實在的把孩子交給他人照顧感到多少煩惱？ |
| 9. | 在過往一星期，你因他人缺乏對食物敏感嚴重性的認知而產生的苦惱感到多少煩惱？ |
| 10. | 在過往一星期，你因孩子患有食物敏感引起的負擔所帶來的憂愁感到多少煩惱？ |
| 11. | 在過往一星期，你因孩子患有食物敏感而對他／她上學、露營、日間自我照顧或其他集體活動感到多少煩惱？ |
| 12. | 在過往一星期，你因孩子患有食物敏感而關心他／她的健康感到多少煩惱？ |
| 13. | 在過往一星期，你憂慮如孩子對食物產生敏感反應時未能幫助他／她感到多少煩惱？ |
| 14. | 在過往一星期，你因孩子患有食物敏感而憂慮他／她未能正常成長感到多少煩惱？ |
| 15. | 在過往一星期，你因孩子患有食物敏感而關心他／她的營養感到多少煩惱？ |
| 16. | 在過往一星期，你因孩子患有食物敏感而關心他／她在進食時接近他人的問題感到多少煩惱？ |
| 17. | 在過往一星期，你對思想孩子產生食物敏感反應的想法所帶來的驚慌感到多少煩惱？ |
