# Supplementary material for: Translation, cross‐cultural adaptation and validation of the traditional Chinese Food Allergy Quality of Life‐Parental Burden questionnaire into simplified Chinese for use in mainland China
Source: Nurs Open. 2023 May 11;10(8):5627–37. doi: 10.1002/nop2.1807 (PMC10333859; doi:10.1002/nop2.1807)
Supplement: Supplementary file 2 — Table S2 [file NOP2-10-5627-s003.docx]

**Table S2** The Final Simplified Chinese FAQL-PB (in simplified Chinese).

| 条目 | （1=没有烦恼；2=些微烦恼；3=少许烦恼；4=有些烦恼；5=颇大烦恼；6=相当烦恼；7=严重烦恼） |
| --- | --- |
| 1. | 如果你和你的家庭计划假期，你所选择的假期会因孩子患有食物过敏而有多少限制？ |
| 2. | 如果你和你的家庭计划去餐馆用餐，你所选择的餐馆会因孩子患有食物过敏而有多少限制？ |
| 3. | 如果你和你的家庭计划参加他人举办的包含食物的社交活动（如派对、假期等），你因孩子患有食物过敏而对能够参加这些包含食物的社交活动有多少限制？ |
| 4. | 在过往一星期，你因孩子患有食物过敏而对需要额外时间准备饭菜（如阅读标签、额外购物时间、准备额外饭菜等）感到多少烦恼？ |
| 5. | 在过往一星期，你因孩子患有食物过敏而对和孩子外出需要做的特别预备措施感到多少烦恼？ |
| 6. | 在过往一星期，你因孩子患有食物过敏而产生的紧张不安感到多少烦恼？ |
| 7. | 在过往一星期，你对孩子可能不能够克服食物过敏而感到多少烦恼？ |
| 8. | 在过往一星期，你因孩子患有食物过敏而可能或的确要把孩子交给他人照顾感到多少烦恼？ |
| 9. | 在过往一星期，你因他人缺乏对食物过敏严重性的认知而产生的苦恼感到多少烦恼？ |
| 10. | 在过往一星期，你因孩子患有食物过敏引起的负担所带来的忧愁感到多少烦恼？ |
| 11. | 在过往一星期，你因孩子患有食物过敏而对他/她上学、露营、日间自我照顾或其他集体活动感到多少烦恼？ |
| 12. | 在过往一星期，你因孩子患有食物过敏而关心他/她的健康感到多少烦恼？ |
| 13. | 在过往一星期，你忧虑如果孩子对食物产生过敏反应时未能帮助他/她而感到多少烦恼？ |
| 14. | 在过往一星期，你因孩子患有食物过敏而忧虑他/她未能正常成长感到多少烦恼？ |
| 15. | 在过往一星期，你因孩子患有食物过敏而关心他/她的营养感到多少烦恼？ |
| 16. | 在过往一星期，你因孩子患有食物过敏而关心他/她在进食时接近他人的问题感到多少烦恼？ |
| 17. | 在过往一星期，你对想到孩子将会产生食物过敏反应时所带来的惊慌感到多少烦恼？ |
