# Supplementary material for: Translation, cross‐cultural adaptation and validation of the traditional Chinese Food Allergy Quality of Life‐Parental Burden questionnaire into simplified Chinese for use in mainland China
Source: Nurs Open. 2023 May 11;10(8):5627–37. doi: 10.1002/nop2.1807 (PMC10333859; doi:10.1002/nop2.1807)
Supplement: Supplementary file 3 — Table S3 [file NOP2-10-5627-s001.docx]

**Table** **S3** The computed content validity index of the 17 items of the SC-FAQL-PB.

| Category | I-CVI | S-CVI/Ave | S-CVI/UA |
| --- | --- | --- | --- |
| Item1 | 1.00 | N/A | N/A |
| Item 2 | 1.00 | N/A | N/A |
| Item 3 | 1.00 | N/A | N/A |
| Item 4 | 1.00 | N/A | N/A |
| Item 5 | 1.00 | N/A | N/A |
| Item 6 | 1.00 | N/A | N/A |
| Item 7 | 1.00 | N/A | N/A |
| Item 8 | 1.00 | N/A | N/A |
| Item 9 | 1.00 | N/A | N/A |
| Item 10 | 0.83 | N/A | N/A |
| Item 11 | 1.00 | N/A | N/A |
| Item 12 | 1.00 | N/A | N/A |
| Item13 | 1.00 | N/A | N/A |
| Item 14 | 1.00 | N/A | N/A |
| Item 15 | 1.00 | N/A | N/A |
| Item 16 | 1.00 | N/A | N/A |
| Item 17 | 1.00 | N/A | N/A |
| The SC-FAQL-PB | N/A | 0.99 | 0.94 |

Note: I-CVI: item of content validity index; S-CVI/Ave: the average S-CVI; S-CVI/UA: universal agreement.
